# Serologic Assessment of Possibility for MERS-CoV Infection in Equids

**DOI:** 10.3201/eid2101.141342

**Published:** 2015-01

**Authors:** Benjamin Meyer, Ignacio García-Bocanegra, Ulrich Wernery, Renate Wernery, Andrea Sieberg, Marcel A. Müller, Jan Felix Drexler, Christian Drosten, Isabella Eckerle

**Affiliations:** University of Bonn Medical Centre, Bonn, Germany (B. Meyer, A. Sieberg, M.A. Müller, C. Drosten, J.F. Drexler, I. Eckerle);; Universidad de Córdoba, Córdoba, Spain (I. García-Bocanegra);; Central Veterinary Research Laboratory, Dubai, United Arab Emirates (U. Wernery, R. Wernery)

**Keywords:** Middle East respiratory syndrome, coronavirus, livestock, intermediate host, zoonoses, horse, equid, equine, infection, MERS, MERS-CoV, CoV

**To the Editor:** In 2012, a novel coronavirus termed Middle East respiratory syndrome coronavirus (MERS-CoV) emerged on the Arabian Peninsula; the virus has been responsible for >800 human cases. Recently, evidence of MERS-CoV infection in dromedaries was obtained from the Canary Islands, the Arabian Peninsula, and Africa (*1*–*3*). Viral sequences from dromedaries and from humans infected with MERS-CoV were highly similar, suggesting a prominent role of dromedaries as an animal reservoir of the virus (*4*). However, the serologic assessment of other animal species has been incomplete. Investigations of domestic animal species have been restricted to goats, sheep, and cattle (*3*) and a limited study of horses (n = 3) (*5*). No evidence of recent infection was found in either study.

Whereas most known CoVs have a highly restricted host range in vitro and in vivo, MERS-CoV has been found to infect a broad range of cell cultures derived from Old and New World camelids as well as humans, primates, bats, pigs, and goats (*6*). MERS-CoV uses the receptor dipeptidyl-peptidase-4 (DPP-4) to enter its host cell (*7*). Sequence comparison between the receptor-binding domain of the MERS-CoV spike protein and several mammalian DPP-4 sequences showed a higher percentage identity in the amino acid residues critical for virus entry between human and horse DPP-4 than between human and dromedary DPP-4 (*8*). It has been shown that MERS-CoV can use horse DPP-4 expressed on nonsusceptible cells (*9*), but no data are available on susceptibility of primary horse cells. Therefore, members of the family *Equidae*, which include domestic horses, donkeys, and mules, might be susceptible to MERS-CoV infection. According to the Food and Agricultural Organization of the United Nations (http://faostat.fao.org), >800,000 equids (horses, mules, and donkeys) are present on the Arabian Peninsula, but their role as putative MERS-CoV animal reservoirs has not been investigated. Therefore, we assessed in vitro susceptibility of primary horse cells to MERS-CoV infection and searched for serologic evidence of infection with MERS-CoV in equids originating from Spain and the United Arab Emirates.

Primary cells derived from the kidney of 2 horses (termed PN-R and PFN-R) and an interferon-deficient primate cell line (VeroB4) were infected with MERS-CoV at a multiplicity of infection of 0.5 PFUs. Virus replication was quantified by real-time reverse transcription PCR (MERS-CoV *upE* assay) (*10*) and by plaque assay in Vero cells to confirm the production of infectious virus particles. Both cell lines showed clear cytopathic effects, an increase of viral RNA, and production of infectious virus progeny (Figure, panels A, B).

**Figure:**
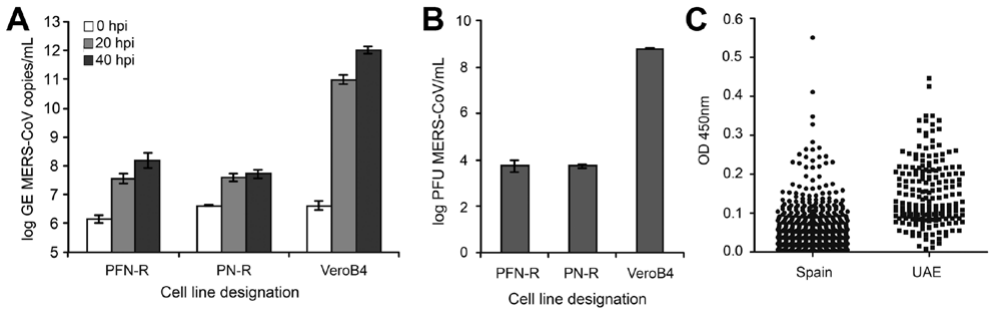
Analysis of the replication of Middle East respiratory syndrome coronavirus (MERS-CoV) in primary horse kidney cell lines and origin of equine serum samples. A, B) Cells were seeded at densities of 2 × 10^5^ cells/mL and infected in triplicate with a multiplicity of infection of 0.5 infectious MERS-CoV units/cell. After incubation for 1 h, cells were washed twice and supernatants were harvested at 0, 20, and 40 h postinfection (hpi). The replication level is given as the log of the genome equivalents (A) or as PFUs (B). Error bars indicate ranges; PF-N and PFN-R indicate the 2 horse cell lines; VeroB4 is an interferon-deficient primate cell line. C) Distribution of optical density (OD) values (450 nm) of equine serum samples originating from Spain or the United Arab Emirates (UAE).

To investigate equids for signs of infection with MERS-CoV, we collected 1,053 serum samples from MERS-CoV–endemic and –nonendemic areas: 192 samples from adult endurance horses from the United Arab Emirates that were collected for routine veterinary purposes; and 861 samples from 697 horses, 82 donkeys, and 82 mules in Spain. Because ELISA optical density (OD) cutoff values for equid serum have not been established, we established a 2-stage algorithm for serologic testing that did not involve the determination of reactivity cutoff values. The screening stage involved testing of all serum samples by using a previously described ELISA with the spike protein S1-domain of MERS-CoV as the test antigen (*4*). The ELISA was adapted for use with horse serum by exchange of the secondary antibody. All serum samples reacted with low to medium OD values (range 0.0–0.55) (Figure, panel C). We then tested the 50 most reactive serum samples (OD range 0.22–0.55) by using recombinant immunofluorescent and microneutralization assays (*1*). These assays are more specific than the ELISA assay and therefore can be used for confirmation. None of the tested serum samples showed reactivity in the recombinant immunofluorescent or microneutralization assays; this finding suggests that no previous exposure of equids to MERS-CoV has occurred in the United Arab Emirates and Spain.

Identifying all potential animal reservoirs is a critical step in controlling zoonotic diseases. Molecular data suggest that horses may be highly susceptible to MERS-CoV because of their high similarity in DPP-4 amino acids at positions critical for binding of the MERS-CoV spike protein (*8*). Our in vitro data confirm the susceptibility of primary horse cells, showing production not only of viral RNA but also of infectious virus progeny, which is a prerequisite for transmission. The lower replication observed in horse cells than in VeroB4 cells may be the result of a difference in the interferon competence of the cells; replication levels in horse cells are comparable to those in bat cells (*6*). Although we did not find evidence for equid infections with MERS-CoV in this study, the general susceptibility on the cell culture level suggests that equids from MERS-CoV–endemic areas, such as Africa and the Arabian Peninsula, should be further investigated for possible infection with MERS-CoV.
